# A Comprehensive App That Improves Tuberculosis Treatment Management Through Video-Observed Therapy: Usability Study

**DOI:** 10.2196/17658

**Published:** 2020-07-31

**Authors:** Xujun Guo, Yarui Yang, Howard E Takiff, Minmin Zhu, Jianping Ma, Tao Zhong, Yuzheng Fan, Jian Wang, Shengyuan Liu

**Affiliations:** 1 Department of Tuberculosis Control and Prevention Shenzhen Nanshan Centre for Chronic Disease Control Shenzhen China; 2 Pathogenomique Mycobacterienne Integree Institut Pasteur Paris France; 3 Laboratorio de Genética Molecular Instituto Venezolano de Investigaciones Cientificas Caracas Venezuela

**Keywords:** tuberculosis, management, video-observed therapy, directly observed therapy, mobile phone

## Abstract

**Background:**

Treatment of pulmonary tuberculosis (TB) requires at least six months and is compromised by poor adherence. In the directly observed therapy (DOT) scheme recommended by the World Health Organization, the patient is directly observed taking their medications at a health post. An alternative to DOT is video-observed therapy (VOT), in which the patients take videos of themselves taking the medication and the video is uploaded into the app and reviewed by a health care worker. We developed a comprehensive TB management system by using VOT that is installed as an app on the smartphones of both patients and health care workers. It was implemented into the routine TB control program of the Nanshan District of Shenzhen, China.

**Objective:**

The aim of this study was to compare the effectiveness of VOT with that of DOT in managing the treatment of patients with pulmonary TB and to evaluate the acceptance of VOT for TB management by patients and health care workers.

**Methods:**

Patients beginning treatment between September 2017 and August 2018 were enrolled into the VOT group and their data were compared with the retrospective data of patients who began TB treatment and were managed with routine DOT between January 2016 and August 2017. Sociodemographic characteristics, clinical features, treatment adherence, positive findings of sputum smears, reporting of side effects, time and costs of transportation, and satisfaction were compared between the 2 treatment groups. The attitudes of the health care workers toward the VOT-based system were also analyzed.

**Results:**

This study included 158 patients in the retrospective DOT group and 235 patients in the VOT group. The VOT group showed a significantly higher fraction of doses observed (*P*<.001), less missed observed doses (*P*<.001), and fewer treatment discontinuations (*P*<.05) than the DOT group. Over 79.1% (186/235) of the VOT patients had >85% of their doses observed, while only 16.4% (26/158) of the DOT patients had >85% of their doses observed. All patients were cured without recurrences. The VOT management required significantly (*P*<.001) less median patient time (300 minutes vs 1240 minutes, respectively) and transportation costs (¥53 [US $7.57] vs ¥276 [US $39.43], respectively; *P*<.001) than DOT. Significantly more patients (191/235, 81.3%) in the VOT group preferred their treatment method compared to those on DOT (37/131, 28.2%) (*P*<.001), and 92% (61/66) of the health care workers thought that the VOT method was more convenient than DOT for managing patients with TB.

**Conclusions:**

Implementation of the VOT-based system into the routine program of TB management was simple and it significantly increased patient adherence to their drug regimens. Our study shows that a comprehensive VOT-based TB management represents a viable and improved evolution of DOT.

## Introduction

Over 10 million people have been reported to develop tuberculosis (TB) in a year globally [1]. Although the incidence of TB in China has been decreasing faster than the rates of global decline, 800,000 TB cases are still being diagnosed annually [2-4], thereby imposing a significant cost on the nation’s health care system. To achieve a high probability of cure with the current treatment regimens, patients with TB must take a combination of drugs for at least 6 months if the strain is sensitive to all antibiotics and for even longer periods if the strain is drug-resistant. If patients stop taking drugs when they feel better or if they miss a significant proportion of their doses, their chances of being cured are reduced. To encourage patient compliance with the treatment regimen, the World Health Organization has a strategy known as the directly observed therapy (DOT), which requires the patients to come to a health post daily to receive their pills so that their pill intakes are monitored. DOT has been credited with improved cure rates in some settings [5,6] but it presents challenges for both patients and the health care workers such that its implementation has become limited [7-10]. DOT imposes a burden on patients in terms of the time needed each day to visit the health post and the consequent absence from home or work responsibilities, the possible cost of transportation, particularly in rural areas, as well as the potential stigma. In addition, the daily administration of medication to all patients with TB is challenging for the health care staff.

Using the technical capabilities of smartphones, several studies have explored alternatives that employ video-observed therapy (VOT), which are less disruptive to the patients’ work and family life, more cost-effective, and improves the patients’ access to doctors [11-13]. The World Health Organization conditionally recommended VOT as an alternative to DOT in 2017, but the evidence was graded as weak because only few randomized controlled trials had been published [14,15]. In the VOT method, the patients either connect via a computer or a smartphone to health care workers who will observe them swallowing the medication in real time [16,17] or alternatively, the patients record videos of themselves taking the pills and then send the videos to the health care workers who can view these videos at their convenience [18].

We developed a comprehensive software for the management of patients with TB by using video monitoring through mobile phones, thereby combining the advantages and the convenience of the VOT method mentioned above. The patients visit their health posts weekly to collect their medications and send videos of themselves taking their pills daily in real time to the health care workers who can review these videos at their convenience. In this study, we evaluate the software for this VOT strategy and its implementation into the routine TB control program of the Nanshan District of Shenzhen, China, and compare the effectiveness of VOT with that of DOT by examining the retrospective data from patients during the year preceding the implementation of the VOT-based program.

## Methods

### Settings

Shenzhen is a rapidly growing metropolis in the south of China that attracts internal migrant workers from all over the country. The Nanshan District is located in the southwest of Shenzhen, with an area of 187.53 square kilometers and a per capita gross domestic product of US $51,000 in 2018 [19], which is the highest for all districts or counties in China. The penetration rate of mobile phones among the residents and workers in Shenzhen is estimated to be over 90%.

### Study Design and Participants

We assessed the effectiveness of the comprehensive TB management system that was implemented in September 2017 as the routine management procedure in the Center for Chronic Disease Control (CCDC) of the Nanshan district of Shenzhen, China. Patients beginning treatment for TB between September 2017 and August 2018 were enrolled into the VOT group, and their data were compared with the retrospective data collected from patients who began TB treatment between January 2016 and August 2017 and were managed with routine DOT. All health care workers involved in the management of patients with TB in the Nanshan District participated in the study, and they had managed patients on DOT before VOT was implemented.

The inclusion criteria were as follows: (1) patients with pulmonary drug-sensitive TB who were treated and managed in the Nanshan CCDC; (2) patients who received a regimen containing only oral medication with fixed-dose combination (FDC) pills at an outpatient facility; and (3) patients who voluntarily provided informed consent to allow their data to be used in research studies. Exclusion criteria were as follows: (1) the presence of other conditions that could compromise the performance of VOT or DOT such as mental disorders, visual, hearing, or speech impairment, or the inability to live on their own; (2) patients who for any reason stopped their anti-TB treatment at the Nanshan CCDC, and (3) patients who did not have a smartphone with an internet connection or a family member with a smartphone and internet connection who could help them.

### Comprehensive TB Management System

**Figure 1:**
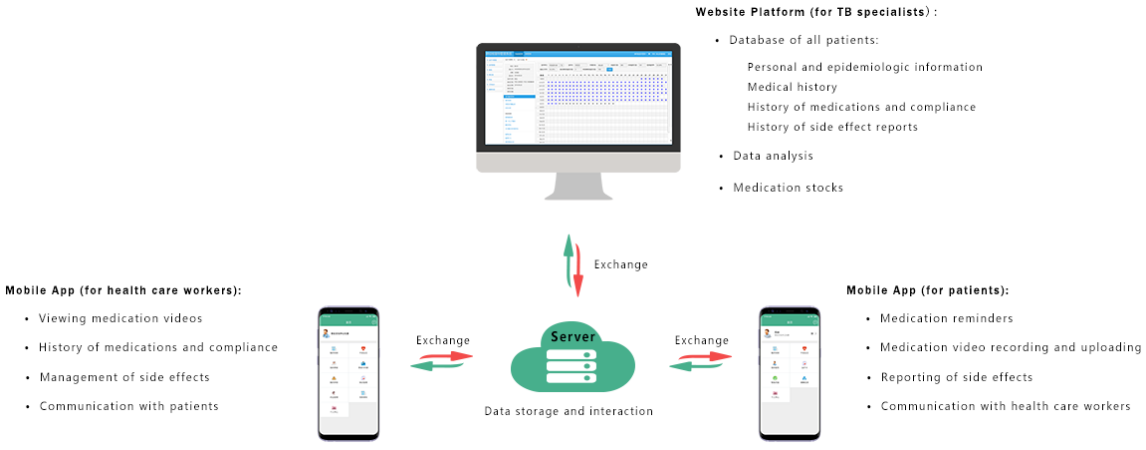
Schematic representation of the data collection and transmission on the comprehensive tuberculosis management system. TB: tuberculosis.

The software for the comprehensive TB management system was independently developed by the Nanshan CCDC. It consists of an app loaded onto the mobile phones of both patients and the medical personnel and a web-based monitoring platform. This app can be used in smartphones with Android or iPhone operating systems, but it is currently available only in Mandarin (Figure 1). This app has multiple functions, including an easy interface to report the side effects, reminders for scheduled medical appointments during treatment, and quantity management of the medicines for the health posts. The patients in the VOT group received training for the following modules in the app: setting up an account, logging into the account, setting the time for their medication reminder, recording and uploading a video, reporting side effects, and communicating with a health care worker. When the patient logs in to the app, a daily pop-up message asks if they notice any adverse reactions, with a selection of options concerning their symptoms and duration and an empty space to describe the problem in detail. Patients can also log into the app at any time to report adverse reactions. If there are no adverse reactions, the patient ignores this pop-up message. This app includes a daily alarm set by the patients based on their own preferences, which reminds them to take their medication. The patients takes a video of them swallowing the pills, which is uploaded in real time to the app server. Patients cannot store the video to upload at a later time. The health care workers assigned to monitor each patient can view the videos at their convenience. If a patient does not send a video for a whole day, after midnight, the app shows a pop-up message on the patient’s phone reminding him/her to take the medication and upload the video. The health care worker for each patient also receives a message that the patient did not upload the video and the health care worker will contact the patient on the following day through the app or by a phone call to remind him/her to take the medication and send the video.

### DOT and VOT procedures

Treatment management with DOT was performed according to the current clinical practice. Most patients with TB in Nanshan are prescribed pills containing FDC of more than one drug per pill, and the patients are instructed to come daily to the community health service center (CHSC) to be observed while taking their medication. The patients requiring nonstandard doses are prescribed pills with the individual drugs that must be taken 2 or 3 times a day. These patients obtain their medicines every 15 days and self-administer these medicines at home. Of the 158 patients in the DOT group who began taking FDC pills once a day at the CHSC, 56 patients had their therapeutic regimen changed to pills with individual drugs, usually because of adverse effects, and the period during which they did not take the FDC pills once daily was excluded from the study.

Patients on VOT management were prescribed FDC drugs and they went to the CHSC every 7 days to receive their medication. Of the 235 patients in the VOT group, 31 patients changed their therapeutic regimen from FDC pills to individual drug pills, and only the period when the patients took the FDC pills once a day was evaluated.

Patients on DOT who failed to visit the health posts daily and patients on VOT who did not send a video were telephoned by health care workers the next day to ask why they missed sending the video and to remind them to take medication. If they could not be telephoned successfully, the health care workers would visit their home within 7 days. If the patient in the VOT group did not upload a video for 3 consecutive days, they were advised to transfer to DOT management. Several patients on DOT adamantly refused to come to the health post daily and were allowed to take their medications at home unobserved.

After successfully finishing the therapy, patients were advised to return to the Nanshan CCDC every 6 months to undergo chest radiography and a sputum smear test for investigation of a recurrence. If they did not return to the CCDC, they would be telephoned and asked to go to the clinic or to report the results of the follow-up tests that they might have done elsewhere. Moreover, periodically, the Nanshan CCDC searched for new diagnostic results of the patients who had finished their TB treatment on the web-based TB information management system set up by the National Center for TB Control and Prevention. All diagnosed TB cases were required to be listed in this registry.

A total of 66 trained health care workers at 66 CHSCs were involved in viewing the videos on the app and briefly seeing the patients when they collected their medications each week. In addition, the patients routinely visited a TB specialist at the Nanshan CCDC every 2 weeks for the first 2 months of treatment and every month for the remainder of the treatment—normally 4 additional months—and blood and urine specimens were obtained for routine tests during these visits. In these visits, the specialists asked the patients whether they missed their doses in order to check the validity of the DOT or VOT records. The TB specialists also periodically reviewed the information provided by the app on the medication stocks. Follow-up sputum smears for microscopy were taken after 2, 3, 5, and 6 months of treatment. Chest radiography images were obtained every 1 or 2 months.

### Data Collection

Responding to questionnaires before and after treatment is the standard procedure for all patients with TB. These questionnaires were handwritten by the patients in the DOT group and electronically completed by the patients in the VOT group. The questionnaire consisted of questions on the sociodemographic variables. After the patients completed the treatment, they were asked to assess their acceptance and satisfaction of their treatment management as well as their incurred costs. The data on compliance and clinical results were collected from handwritten records of the patients on DOT and from the comprehensive TB management system records for the patients on VOT. All patient information was made irreversibly anonymous. After the implementation of the project, we asked 66 health care workers to complete an electronic questionnaire on their attitudes toward patient management with DOT and VOT.

Treatment adherence was measured by the number of missed medications, the number of times the patient discontinued their medications for 3 or more continuous days, and the fraction of doses observed, which is the number of the directly observed or video-observed doses divided by the total number of doses expected to be taken during the course of the treatment.

Patients were asked about the time required to go from their point of departure to the CHSC to obtain their medications, the travel costs, and how long the they typically spent in the CHSC to obtain their medicines and see a health care worker. The total number of trips to the CHSC during the course of treatment was collected from the patient records. The time and costs for single visits were then multiplied by the total number of trips to the CHSC to obtain the total time and total costs for the patients to obtain their medications. The median cost during the VOT study period was ¥7 (US $1).

### Statistical Analysis

We used Kruskal-Wallis test, chi-square test, and Fisher’s exact test within SPSS version 19.0 (IBM Corp) to evaluate the possible differences between the DOT and VOT groups in the sociodemographic characteristics, clinical features, treatment adherence, patient time and costs, positive sputum smear findings, and patient and health care worker satisfaction.

## Results

### Patient Characteristics

This study initially consisted of 393 cases of rifampicin-sensitive pulmonary TB, with 158 patients in the DOT group and 235 patients in the VOT group. There were no statistical differences between the 2 groups in any of the clinical or sociodemographic parameters recorded (*P*>.05) (Table 1 and Table 2). Of the 290 patients initially registered for TB treatment with FDC pills at the Nanshan CCDC between September 2017 to August 2018, only 2.1% (6/290) were excluded from the study because they or their family members lacked a smartphone with an internet connection. Another 49 patients were excluded because they stopped their treatment in Nanshan for reasons including moving out of Shenzhen, stopping their treatment on their own, or because it was no longer possible to contact them. The mean time for learning to use the app was 5.7 (SD 6.6) minutes.

### Validity and Utility of the TB Management Systems

#### Medication Adherence

The fraction of the doses observed in the VOT group was significantly higher than that observed in the DOT group (*P*<.001), with 63.0% (148/235) versus 4.4% (7/158) having ≥95% of their doses observed, respectively (Table 3). The VOT group also had significantly less missed observed doses *(P*<.001) and discontinuations (*P*=.002) than the DOT group. In the DOT group, 55.1% (87/158) of the patients had less than 50% of their doses observed at the CHSC, but 74.1% (117/158) reported no missed doses. This discrepancy is because many of the patients in the DOT group often refused to come to the CHSC daily and were given medications to self-administer at home, which they reported to have taken. Similarly, in the VOT group, only 63.0% (148/235) of the patients had ≥95% of their expected doses observed and 91.1% (214/235) of the patients reported no missed doses. The striking difference was that 79.1% (186/235) of the VOT patients had ≥85% of their doses observed, while only 16.4% (26/158) of the DOT patients had ≥85% of their doses observed. Five patients in the VOT group failed to send a video for at least three consecutive days and were advised to transfer to DOT management, but all of them requested to be allowed to remain on VOT (Table 3).

**Table 1:** Sociodemographic characteristics of the patients in the DOT^a^ and VOT^b^ groups.

Patient demographics		DOT group (n=158), n (%)	VOT group (n=235), n (%)	*P* value
**Gender**				.28
	Male	102 (64.6)	139 (59.1)	
	Female	56 (35.4)	96 (40.9)	
**Ethnicity**				.06
	Han nationality	157 (99.4)	225 (95.7)	
	Others	1 (0.6)	10 (4.3)	
**Occupation**				.59
	Food and beverage industry	3 (1.9)	3 (1.3)	
	Cadre staff	78 (49.4)	134 (57.0)	
	Workers	27 (17.1)	33 (14.0)	
	Domestic and unemployed	33 (20.9)	33 (14.0)	
	Teachers	1 (0.6)	1 (0.4)	
	Retired personnel	2 (1.3)	4 (1.7)	
	Students	9 (5.7)	17 (7.2)	
	Other	5 (3.2)	10 (4.3)	
**Education level**				.31
	Junior high school and below	29 (18.4)	39 (16.6)	
	High school	65 (41.1)	115 (48.9)	
	Undergraduate and above	64 (40.5)	81 (34.5)	
**Social health insurance status**				.91
	Shenzhen Medical Insurance 1st	64 (40.5)	87 (37.0)	
	Shenzhen Medical Insurance 2nd	38 (24.1)	61 (26.0)	
	Shenzhen Medical Insurance 3rd	13 (8.2)	26 (11.0)	
	Medical insurance outside Shenzhen	15 (9.5)	19 (8.1)	
	No medical insurance	16 (10.1)	22 (9.4)	
	Other	12 (7.6)	20 (8.5)	
**Was there a stable residence during the period of treatment?**				.98
	No	10 (6.3)	15 (6.4)	
	Yes	148 (93.7)	220 (93.6)	
**Annual family total income (**¥**), (~Max USD)**				.47
	<10,000 ($1,430)	1 (0.6)	2 (0.9)	
	10,000-99,999 ($14,300)	64 (40.5)	104 (44.3)	
	100,000-199,999 ($28,600)	49 (31.0)	67 (28.5)	
	200,000-299,999 ($42,900)	32 (20.3)	34 (14.5)	
	300,000-399,999 ($57,200)	7 (4.4)	13 (5.5)	
	≥400,000	5 (3.2)	15 (6.4)	
**Age (years)**				.22
	<25	39 (24.7)	75 (31.9)	
	25-44	99 (62.7)	139 (59.1)	
	45-64	17 (10.8)	20 (8.5)	
	≥65	3 (1.9)	1 (0.4)	

^a^DOT: directly observed therapy.

^b^VOT: video-observed therapy.

**Table 2:** Clinical features of the patients in the DOT^a^ and VOT^b^ groups.

Clinical features		DOT group (n=158), n (%)	VOT group (n=235), n (%)	*P* value
**Abnormal chest radiographs**				N/A^c^
	Yes	158 (100.0)	235 (100.0)	
	No	0 (0)	0 (0)	
**Cavity**				.28
	Yes	36 (22.8)	43 (18.3)	
	No	122 (77.2)	192 (81.7)	
**Registration classification**				.65
	New patients	157 (99.4)	232 (98.7)	
	Patients with recurrence	1 (0.6)	3 (1.3)	
**Symptoms**				.16
	Night sweat	1 (0.6)	0 (0)	
	Cough for less than 2 weeks	14 (8.9)	22 (9.4)	
	Cough for more than 2 weeks	34 (21.5)	57 (24.3)	
	Cough sputum for less than 2 weeks	3 (1.9)	0 (0)	
	Cough sputum for more than 2 weeks	1 (0.6)	0 (0)	
	Hemoptysis or blood sputum	7 (4.4)	17 (7.2)	
	Chest tightness	0 (0)	3 (1.3)	
	Chest pain	10 (6.3)	19 (8.1)	
	Asymptomatic	88 (55.7)	117 (49.8)	
**Sputum smears at the time of diagnosis**				.91
	Negative	125 (79.1)	194 (82.6)	
	2-3 bacilli/300 fields of view	0 (0)	1 (0.4)	
	4-10 bacilli/300 fields of view	3 (1.9)	2 (0.9)	
	3-9 bacilli/100 fields of view	7 (4.4)	11 (4.7)	
	1-9 bacilli/10 fields of view	8 (5.1)	9 (4.3)	
	1-9 bacilli/field of view	6 (3.8)	7 (3.0)	
	≥10 bacilli/field of view	9 (5.7)	11 (4.7)	
**Sputum cultures at the time of diagnosis**				.37
	Positive	72 (45.6)	118 (50.2)	
	Negative	86 (54.4)	117 (49.8)	

^a^DOT: directly observed therapy.

^b^VOT: video-observed therapy.

^c^Not applicable: Chi-square test or Fisher’s exact test could not be used when 2 groups are compared with all cases representing the same results.

**Table 3:** Compliance with medication and adverse event management of the patients.

Characteristics		DOT^a^ group (n=158), n (%)	VOT^b^ group (n=235), n (%)	*P* value* *
**Fraction of the doses observed^c^ (%)**				<.001
	<50	87 (55.1)	7 (3.0)	
	50-74	22 (13.9)	19 (8.1)	
	75-84	23 (14.9)	23 (9.8)	
	85-89	10 (6.3)	17 (7.2)	
	90-94	9 (5.7)	21 (8.9)	
	≥95	7 (4.4)	148 (63.0)	
**Reported missed medication doses^d^**				<.001
	0	117 (74.1)	214 (91.1)	
	1-5	35 (22.2)	19 (8.1)	
	6-10	5 (3.2)	0 (0)	
	>10	1 (0.6)	1 (0.4)	
**Number of times the medication was discontinued for ≥3 days^e^**				.002
	0	147 (93.0)	230 (97.9)	
	1	10 (6.3)	2 (0.9)	
	2	0 (0)	2 (0.9)	
	4	0 (0)	1 (0.4)	
	5	1 (0.6)	0 (0)	
**Adverse events reported by patients**				.13
	Yes	30 (19.0)	60 (25.5)	
	No	128 (81.0)	175 (74.5)	
**Number of adverse events reported by patients**				.22
	0	128 (81.0)	175 (74.5)	
	1	26 (16.5)	54 (23.0)	
	2	3 (1.9)	6 (2.6)	
	3	1 (0.6)	0 (0)	
**Suspension due to medical advice**				.19
	Yes	33 (20.9)	37 (15.7)	
	No	125 (79.1)	198 (84.3)	
**Number of suspensions due to medical advice**				.22
	0	125 (79.1)	198 (84.3)	
	1	33 (20.9)	35 (14.9)	
	2	0 (0)	1 (0.4)	
	4	0 (0)	1 (0.4)	

^a^DOT: directly observed therapy.

^b^VOT: video-observed therapy.

^c^The number of observed doses divided by the total number of doses expected to be taken. The numerator includes either directly observed or video-observed doses, excluding those obtained by self-report. The denominator includes the self-administered medications; it does not include periods when the treatment was suspended based on medical advice because of side effects.

^d^Missed doses reported by the patients, as the patients on VOT could have taken their medications but they may have forgotten to record a video or they may have self-administered DOT medications at home; therefore, the patients could have taken the reported doses more than the ones observed by the health care workers.

^e^The times when patients missed their doses for at least three consecutive days on their own, not including the occasions when medication was suspended owing to medical advice.

#### Clinical Results

At the time of the diagnosis, 33 of the 158 patients in the DOT group showed positive sputum smear findings, while 40 showed smear-negative and culture-positive findings. Of the 235 patients in the VOT group, 40 showed positive smear findings and 82 showed negative smears but positive culture findings. Because clinical improvement was followed based upon sputum smear findings, only patients with positive smear findings could be compared to monitor the treatment success by observing for the clearance of the bacilli from the sputum. In most patients, the bacilli were cleared from the sputa after 2 months of therapy, but more patients in the DOT group required 3 months (15%, 5/33 vs 5%, 2/40) or 5 months (3%, 1/33 vs 0%, 0/40) for their sputum smear findings to be negative than those in the VOT group, respectively, although the difference was not statistically significant (*P*=.17). All patients showed negative smears by the end of their 6 months of treatment, and no recurrences were recorded in either group after at least six months of follow-up.

#### Management of the Side Effects

Adverse events were reported to the health care workers by 19.0% (30/158) of the patients in the DOT group and 25.5% (60/235) patients in the VOT group (Table 3). Medication was suspended in 20.9% (33/158) of the patients in the DOT group and 15.7% (37/235) of the patients in the VOT group, most commonly for abnormal results for liver function tests and for rash, itching, or hives. None of the differences between the 2 groups related to side effects was statistically significant (*P*>.05), but significantly more patients in the DOT group (*P*<.001) than those in the VOT group had their therapeutic regimen changed (35.4%, 56/158 vs 13.2%, 31/235, respectively).

### Patient Transportation Time and Expenditures

#### Time Required

After completing the treatment, the patients were given questionnaires about various aspects of their experiences during the treatment. These questionnaires were completed by 82.9% (131/158) of the patients in the DOT group and 100.0% (235/235) of the patients in the VOT group, although not all the participants answered all the questions. Approximately 64.8% (81/125) of the patients in the DOT group and 59.1% (130/220) of the patients in the VOT group walked from their homes or places of work to the CHSC, and the median distance was 1.0 km. The median time for a one-way trip to the CHSC in the DOT group and VOT group was 10 minutes and 15 minutes, respectively, but the difference was not statistically significant (*P*=.25). If all patients complied with the expected number of visits to the CHSC and CCDC, the median total time spent traveling for their daily visits over 6 months would have been about 3600 minutes or 60 hours in the DOT group compared to 720 minutes or 12 hours in the VOT group. The time calculated from the actual median number of visits was much less in the DOT group, that is, 1240 minutes or 21 hours, because 55.1% (87/158) of the patients skipped more than half of the expected daily CHSC visits. In contrast, the median time calculated from the actual visits to the CHSC in the VOT group was 300 minutes or 5 hours (Table 4).

**Table 4:** Transportation time to the community health service center and the transportation costs incurred by the patients.

Transportation time and costs	DOT^a^ group, median (IQR)	VOT^b^ group, median (IQR)	*P* value
One-way distance (km)	1 (0.55, 2)	1 (1, 2)	.01
Time for one-way trip (min)	10 (5, 30)	15 (10, 20)	.25
Cumulative total time for the trips (min)	1240 (540, 3041)	300 (140, 720)	<.001
One-way cost of transportation (¥)^c^	2 (2, 5)	2 (1, 5)	.32
Cumulative total cost of transportation (¥)	276 (115, 552)	53 (25.5, 208)	<.001

^a^DOT: directly observed therapy.

^b^VOT: video-observed therapy.

^c^¥1=US $0.14

#### Transportation Costs

Of the patients reporting the details of their visits to the CHSC, 35.2% (44/125) in the DOT group and 40.9% (90/220) in the VOT group could not walk to the CHSC and were given funds for public transportation. The median transportation cost of a round trip visit to the CHSC, according to the patients who completed the questionnaire, was about ¥4 (US $0.57), resulting in an estimated total transportation cost of ¥96 (US $13.71) for the VOT patients compared with ¥720 (US $102.86) for the DOT patients. Although the actual costs were less, especially because most DOT patients self-administered many of their doses at home, the VOT patients still spent much less on transportation than the DOT patients (¥53 [US $7.57] vs ¥276 [US $39.43], respectively; *P*<.001) (Table 4).

### Acceptance and Satisfaction

#### Satisfaction with the Management Methods

The patients in the DOT group were evenly divided about the ease of completing their drug regimen with half (49.6%, 65/131) regarding it as easy or very easy and half (50.4%, 66/131) regarding it as difficult or very difficult. In contrast, 93.2% (219/235) of the patients in the VOT group described it as easy or very easy, 6.8% (16/235) as difficult, and none described this method as very difficult. In the VOT group, 80.4% (189/235) thought that the use of the app could reduce the number of missed doses, while only 32.1% (42/131) in the DOT group thought that the daily visits would reduce the missed doses. Of the DOT patients, 40.5% (53/131) were satisfied or very satisfied, while 35.1% (46/131) were not satisfied or were very dissatisfied with their treatment management, and 71.8% (94/131) referred a self-administered scheme to the daily visits. Of the patients on VOT, 81.3% (191/235) were satisfied or very satisfied, with only 3.4% (8/235) not satisfied, and 81.3% (191/235) indicated they would prefer this method of treatment (Table 5).

The majority of the patients in both the groups (64.9%, 85/131 in DOT and 56.6%, 133/235 in VOT) felt that there was no violation of their privacy, but a significant minority (25.2%, 33/131 in DOT and 37%, 87/235 in VOT) thought that there were occasional violations, and privacy violations were felt to be often or always by 9.9% (13/131) of the patients in the DOT group and 6.4% (15/235) of the patients in the VOT group (Table 5).

Of the 66 health care workers who completed the survey, 24% (16/66) were males with a mean age of 36.20 (SD 7.19) years; 30 were nurses, 24 were physicians, 7 were pharmacists, 2 were public health doctors; and there was a laboratory worker, a social worker, and a cashier. All had worked with both the DOT and VOT methods. Most health care workers (92%, 61/66) thought that the VOT method was convenient to manage patients with TB, as it could improve the management of the patients with TB (86%, 57/66) and they preferred the VOT method over the DOT method (85%, 56/66) (Table 5).

**Table 5:** Measurements of the satisfaction of the patients and the health care workers with the DOT^a^ or VOT^b^ methods.

Questions		DOT group (n=131), n (%)	VOT group (n=235), n (%)	*P* value	Health care workers (n=66), n (%)
**What do you think of going to the CHSC^c^ daily or of using an app to upload a video daily?**				<.001	
	Very easy	25 (19.1)	122 (51.9)		N/A^d^
	Easy	40 (30.5)	97 (41.3)		N/A
	Little difficult	52 (39.7)	16 (6.8)		N/A
	Very difficult	14 (10.7)	0 (0)		N/A
**What is the frequency of the perceived privacy violation going to the CHSC or taking and uploading a video?**				.12	
	Always	6 (4.6)	7 (3.0)		N/A
	Often	7 (5.3)	8 (3.4)		N/A
	Occasionally	33 (25.2)	87 (37.0)		N/A
	No	85 (64.9)	133 (56.6)		N/A
**Does your management method reduce the number of missed medications?**				<.001	
	Yes	42 (32.1)	189 (80.4)		N/A
	No	89 (67.9)	46 (19.6)		N/A
**Which management method do you prefer?**				<.001	
	Their current management method	37 (28.2)	191 (81.3)		N/A
	Self-administered medication	94 (71.8)	44 (18.7)		N/A
**Are you satisfied with the prescribed management method?**				<.001	
	Very satisfied	23 (17.6)	98 (41.7)		N/A
	Fairly Satisfied	30 (22.9)	93 (39.6)		N/A
	Just Satisfied	32 (24.4)	36 (15.3)		N/A
	Not satisfied	35 (26.7)	8 (3.4)		N/A
	Very dissatisfied	11 (8.4)	0 (0)		N/A
**Was the VOT app convenient for managing patients with TB^e^?**				N/A	
	To a large extent	N/A	N/A		61 (92)
	Rarely	N/A	N/A		4 (6)
	No	N/A	N/A		1 (2)
**Could the VOT app improve the standard management of patients with TB?**				N/A	
	To a large extent	N/A	N/A		57 (86)
	Rarely	N/A	N/A		8 (12)
	No	N/A	N/A		1 (2)
**Which management method did the health care workers prefer?**				N/A	
	VOT	N/A	N/A		56 (85)
	DOT	N/A	N/A		1 (2)
	No special preference	N/A	N/A		9 (14)

^a^DOT: directly observed therapy.

^b^VOT: video-observed therapy.

^c^CHSC: community health service center.

^d^Not applicable.

^e^TB: tuberculosis.

#### Acceptance of the App

In the VOT group, 96.6% of the patients (227/235) could record the medication administration video by themselves, and the same proportion of patients thought that uploading the video was simple or very simple. Eight patients needed the help of their family to record and upload the videos; 88.9% (209/235) of the patients reported that problems using the app or in uploading the videos occurred less than 10% of the time (Table 6).

The health care workers reported that the duration of the videos was approximately 10-30 seconds each. The mean, median, maximum, and minimum number of patients that each health care worker managed were 3.6, 2, 1, and 19, respectively. The majority (89%, 59/66) of the health care workers were satisfied with the app interface, the time required to work with the app (85%, 56/66), and the quality of the video recordings (88%, 58/66). For the majority of the health care workers (96%, 63/66), problems with the app occurred less than 10% of the time.

**Table 6:** Acceptance of the patients on VOT^a^ and health care workers with the use of the app.

Questions		Patients on VOT (n=235), n (%)		Health care workers (n=66), n (%)	
**Could you record the medicine administration video by yourself?**					
	Yes		227 (96.6)		N/A^b^
	No		8 (3.4)		N/A
**What is the degree of difficulty in using the app to upload the medicine administration video?**					
	Very simple		143 (60.9)		N/A
	Simple		84 (35.7)		N/A
	A little difficult		8 (3.4)		N/A
**How frequently (%) did the app cause problems during the course of the treatment?**					
	0		57 (24.3)		18 (27)
	<10		152 (64.7)		45 (68)
	<50		21 (8.9)		2 (3)
	≥50		5 (2.1)		1 (2)
**Are you satisfied with the app interface?**					
	Very satisfied		153 (65.1)		32 (48)
	Satisfied		80 (34.0)		27 (41)
	Not satisfied		2 (0.9)		7 (11)
**Are you satisfied with the time spent on the medicine administration videos?**					
	Very satisfied		156 (65.1)		37 (56)
	Satisfied		77 (32.76)		19 (29)
	Not satisfied		2 (0.9)		10 (15)
**Are you satisfied with the quality of the video recording?**					
	Very satisfied		N/A		36 (55)
	Satisfied		N/A		22 (33)
	Not very satisfied		N/A		8 (12)

^a^VOT: video-observed therapy.

^b^Not applicable.

## Discussion

### Principal Findings

In this study, we compared the management of patients with TB with a comprehensive VOT management system and the management of patients on DOT by using retrospective data from the year preceding the implementation of VOT. In terms of the number of medication doses observed, missed doses, patient time required and transportation costs, ease of use, and acceptance by patients and medical staff, the VOT management system performed better than the DOT management system.

On the basis of the findings for smears and cultures, 46.2% (73/158) of the DOT patients and 51.9% (122/235) of the VOT patients had a bacteriologically confirmed diagnosis of TB, which is higher than the overall rate of bacteriologically confirmed diagnosis of TB cases of 37% for China [20]. However, the proportion of patients who showed positive smears was low: 20.9 % (33/158) of the patients in the DOT group and 17.4% (41/235) of the patients in the VOT group. Because therapy was only followed on the basis of the monthly sputum smears, only this small proportion of patients could be followed up for the clinical results. In these patients with positive smears, there was no statistical difference (*P*=.17) in the clinical results, and all the patients showed negative smears after completing 6 months of treatment. However, 95% (38/40) of the patients on VOT had negative smears at 2 months and 100% (40/40) of the patients on VOT showed negative smears at 3 months, while 82% (27/33) of the patients on DOT showed negative smears at 2 months, 97% (32/33) showed negative smears at 3 months, and 1 patient on DOT showed a positive smear at 5 months.

Even in cities with local health posts that are close to the residence of most patients, daily DOT visits can interfere with the responsibilities at home and at work. Although DOT management requires patients come to the health post daily to be observed while taking their medications, many patients refused to come every day and they were given medications to take home, which they generally reported as faithfully self-administered. While we suspected that compliance with DOT might be a problem in China, and perhaps also in other places [21], we did not expect to find that only 1 out of 6 (16.4%) DOT patients had >85% of their doses observed. In contrast, 4 out of 5 (79.1%) of the VOT patients had >85% of their doses observed. Our study also found that the patient costs for transportation and patient time required for health post visits were considerably decreased with VOT, which likely contributed to the success and acceptance of VOT.

DOT can also be a burden on the health care system [22]. In the health care system of the Nanshan district, each health care worker had few patients with TB, but the health care workers had a heavy work burden because they also routinely cared for patients with 13 other conditions, including diabetes, hypertension, and mental illness, and they are involved in maternal and child health care. It was thus not surprising that they were appreciative of the management app that reduced patient visits, thereby saving them time. Similarly, in the typical settings of countries with a high TB burden, wherein overburdened health care workers are responsible for many patients on TB treatment, the VOT app should produce considerable savings in time and effort for the health care staff.

An additional advantage of the comprehensive TB management system is that it automatically monitors the number of patients on therapy, the medication inventories, the clinical results, the incidence of adverse events, and patient compliance in real time. Noncompliant patients or patients with side effects can be identified without delay and they can be contacted. The system thus avoids the need to collect records from various sources or databases so that both patient care and record keeping are more efficient and require less work by the health care system [22-26].

### Limitations

This study had the following limitations. In this study, only patients who took FDC pills once a day were included and patients who took pills more than once a day were excluded. However, if patients need to take a medication more than once a day at home, it should be feasible to send 2 or 3 videos daily, and we plan to implement this possibility in the future. Another limitation is that this study included only patients with drug-sensitive TB, whereas compliance is even more critical for curing patients with drug-resistant TB, who require long treatment regimens with drugs that often have more adverse reactions. VOT would likely be especially useful for these patients [17] with whom DOT has been shown to improve compliance [5].

China is a country wherein VOT could be very successful, especially in large cities like Shenzhen, wherein smartphone penetration is very high and users are accustomed to working with a variety of apps. However, the penetration of mobile phones and facilities with their use may be less in the rural areas of China and in many countries with high TB burden and less resources and infrastructure, and their cost and inadequate internet data connections could be obstacles for VOT management. However, VOT could be the most valuable and cost-effective in these settings because it is in these settings that patients live far from the nearest health posts [27,28]. To function adequately in rural, low-infrastructure settings, the uploading protocol may need to be adapted to work with intermittent internet connectivity while still ensuring that each video has a time stamp and can only be sent once. This was a retrospective study that compared the results of DOT management for TB control in the Nanshan district with the results after the implementation of the comprehensive TB management system with VOT. If the entire study had been carried out in a carefully optimized prospective manner, perhaps the results of all the aspects would have been better than what we observed [29]. However, the results reported here represent what can routinely be expected from VOT implementation in a TB control program because the low proportion of doses that were actually observed with patients on DOT is likely commonplace in many DOT programs in China and perhaps elsewhere [15,30]. We believe that the significant increase in the observed doses with VOT could be a robust finding for programs adopting a comprehensive VOT management system such as that described by us [12,15,18,20].

### Conclusions

After the implementation of a VOT-based comprehensive TB management system into the routine management of patients with TB in the Nanshan District of Shenzhen, the treatment compliance of patients was found to be significantly higher than that with the previous model using DOT. The VOT management system for TB required less patient time, low transportation costs, was easily adopted by the patients, and achieved high rates of satisfaction by both patients and health care workers. The findings of our study suggest that the adoption of the VOT system is feasible for TB control programs and will perform better than using the DOT strategy for the treatment of TB.

## Abbreviations

CCDC: Center for Chronic Disease Control
CHSC: community health service center
DOT: directly observed therapy
FDC: fixed-dose combination
TB: tuberculosis
VOT: video-observed therapy
